# Intergroup contact and Chinese cultural identity: a three-wave study of Taiwanese students enrolled at universities in Mainland China

**DOI:** 10.1080/00049530.2026.2755162

**Published:** 2026-11-04

**Authors:** Qi Xinjie, Zhang Yingying, Lian Rong

**Affiliations:** a Department of Student Affairs, Fujian Chuanzheng Communications College, Fuzhou, China; b School of Psychology, Fujian Normal University, Fuzhou, China

**Keywords:** Intergroup contact, Taiwanese students, Chinese cultural identity, cultural adaptation, openness to experience

## Abstract

**Objective:**

This study addressed inconsistent findings regarding cross-strait integration and the limited application of time-lagged, mechanism-focused approaches. It examined whether intergroup contact was prospectively associated with Chinese cultural identity among Taiwanese students in Mainland China, focusing on cultural adaptation and personality traits.

**Method:**

A six-month, three-wave time-lagged survey was conducted with 286 Taiwanese students at universities in Mainland China. Data were collected at three measurement points using validated self-report instruments to assess intergroup contact, cultural adaptation, openness to experience, and Chinese cultural identity. Baseline identity (T1) was controlled when predicting identity at T3. Mediation and moderation analyses were conducted using PROCESS.

**Results:**

Positive intergroup contact significantly predicted stronger Chinese cultural identity at T3，controlling for baseline levels. Results supported an indirect association through cultural adaptation, but not a full test of longitudinal mediation. Openness to experience did not significantly moderate the association.

**Conclusions:**

Sustained, high-quality intergroup contact was associated with increased cultural identity among Taiwanese youth. The observed temporal ordering is consistent with a possible indirect pathway through cultural adaptation, although the present study does not constitute a full longitudinal test of mediation. Rather than extending intergroup contact theory, the findings offer context-specific support for its applicability in an East Asian setting. Given that the sample was drawn from three universities in two provinces, generalizability is limited. The results further suggest that supportive social and educational environments may be more influential than broad personality traits in fostering integration.

## KEY POINTS

What is already known about this topic:

(1) Positive intergroup contact promotes cultural adaptation and is associated with stronger cultural identity.

(2) Cultural adaptation may be an important psychological process that connects contact quality with identity outcomes.

(3) Openness to experience enhances the quality of cross-group interactions and facilitates cultural integration.

What this topic adds:

(1) This study provides the first longitudinal analysis of Taiwanese students studying in Mainland China, capturing the dynamic evolution of cultural identity under sustained cross-strait contact.

(2) This study highlights the importance of examining intergroup contact in contexts where group boundaries are ambiguous, culturally shared, and politically complex, thereby extending existing theories beyond traditionally distinct group settings.

(3) Results suggest that supportive social and educational environments are more influential than personality traits in promoting cultural identity, offering actionable implications for integration-oriented policy and practice.

## Introduction

As of the end of 2025, the number of Taiwanese students enrolled in universities in Mainland China has reached 12,000, primarily distributed across Beijing, Shanghai, Guangdong, and Fujian. For this group, Chinese cultural identity is reflected in their cognitive orientations toward symbolic, material, and spiritual aspects of Chinese culture, their affective attachments to these elements, and their engagement in related everyday practices (Sun, 2023). Despite sharing strong cultural roots with Mainland China, Taiwanese students in this context exhibit a certain degree of malleability in their political and social identities (H. Lin, 2023). This condition is closely linked to the historically and geographically specific nature of cross-strait relations. Divergent political trajectories and institutional developments have rendered identity expressions increasingly complex (R. Chen et al., 2024), while geographical proximity facilitates interaction and cultural exchange, even as the Taiwan Strait constitutes a boundary that introduces institutional and contextual constraints (Y. Chen, 2022). As a result, shared cultural foundations coexist with fluid and contested identity boundaries, making this population neither comparable to typical cross-cultural groups nor easily situated within clearly bounded intergroup relations.

In light of this context, traditional intergroup contact theory, which is largely developed based on groups with relatively clear boundaries and assumes that contact promotes a gradual shift from outgroup to ingroup identification (Dovidio et al., 2003), shows limitations in explaining the cultural identity formation of Taiwanese students in Mainland China. Empirical findings remain inconsistent: while some studies suggest that cross-context interactions and shared cultural resources (e.g., common traditions and digital media engagement) may strengthen cultural affiliation and emotional attachment (Li, 2025; Lian et al., 2025; Y. Lin & Xu, 2022; Liu, 2021; Zhuang, 2025), others indicate that pre-existing ideological orientations and identity frameworks play a more stable role, such that even frequent contact does not necessarily alter, and may even reinforce, existing identity trajectories (R. Chen et al., 2024; Meng, 2016). This divergence suggests that contact alone is insufficient to account for identity formation in such contexts. Accordingly, an important question arises: to what extent and through what mechanisms does intergroup contact shape Chinese cultural identity among Taiwanese students enrolled in universities in Mainland China under conditions characterized by shared cultural foundations, ambiguous group boundaries, and political complexity? Addressing this question is of particular practical significance, as it provides insight into how identities are negotiated in such contexts, thereby offering implications for fostering constructive interactions and informing similar intergroup relations beyond the Cross-Strait setting.

Accordingly, to address this question, the present study focuses on Taiwanese students enrolled in universities in Mainland China and adopts a longitudinal perspective within a context of sustained intergroup contact. It aims to examine how intergroup contact shapes Chinese cultural identity and to uncover the underlying mechanisms through which this process unfolds. In particular, this study considers the role of cultural adaptation as a key process linking contact experiences to identity outcomes, while also accounting for individual differences in openness to experience as a boundary condition that may shape this relationship. By doing so, this study seeks to advance understanding of how cultural identity is constructed in contexts characterized by shared cultural foundations, ambiguous group boundaries, and political complexity, and to provide empirical insights into the conditions under which intergroup contact facilitates or constrains identity change.

### The impact of intergroup contact on Chinese cultural identity

Intergroup contact refers to direct or indirect interactions between members of different social, cultural, or identity groups, encompassing both actual and symbolic forms of engagement across group boundaries (Paulck et al., 2018). In the present study, intergroup contact is operationalized as the frequency and quality of interactions between Taiwanese students enrolled in universities in Mainland China and their Mainland Chinese peers in both academic and everyday contexts. Such contact may occur through face-to-face communication (e.g., classroom discussions, group projects, and campus activities), interactions in virtual spaces (e.g., social media and online collaboration), or indirect forms of contact mediated by media, social networks, or even imagination (Wang et al., 2022).

According to intergroup contact theory, positive and egalitarian interactions improve intergroup relations through cognitive, affective, and behavioural mechanisms, thereby reducing prejudice and fostering mutual understanding and cooperation (Gaertner et al., 1993; Zhu et al., 2024). Although the relationship between Taiwanese students and Mainland Chinese students does not constitute a prototypical ingroup–outgroup dichotomy, prior research suggests that intergroup contact can still shape individuals’ cognitive and emotional experiences. In particular, contact promotes empathy towards others (Vanman, 2016), which in turn facilitates the perception of shared group membership and contributes to the formation of a common identity (Tassinari et al., 2022). Beyond its influence on intergroup attitudes, intergroup contact also plays a significant role in the formation of cultural identity. Cultural identity can be understood as a multidimensional construct encompassing individuals’ cognitive recognition of, emotional attachment to, and behavioural engagement with a cultural group, reflecting their sense of “who I am” and “to whom I belong” within broader social contexts (Ward & Szabó, 2023). In this study, Chinese cultural identity is operationalized as Taiwanese students’ identification with Chinese culture, including their recognition of shared cultural symbols and values, emotional attachment to Chinese cultural traditions, and participation in related cultural practices (Sun, 2023). In the context of Mainland Chinese universities, Taiwanese students frequently interact with Mainland peers through academic collaboration (e.g., joint coursework and group assignments), daily campus life (e.g., dormitory living and extracurricular activities), and informal social exchanges. When such interactions are characterized by equal status, mutual respect, and shared goals – such as successfully completing academic tasks or engaging in collective activities – students are more likely to develop a deeper understanding of Chinese cultural meanings, strengthen their emotional resonance with cultural traditions, and increase their participation in culturally embedded practices (B. Zhang et al., 2022). Through these processes, intergroup contact facilitates the reconstruction of self-concepts and promotes identification with a broader Chinese cultural community. This mechanism is supported by converging empirical evidence across multiple methodological approaches. Qualitative research shows that positive intergroup contact can drive cultural identity development by reshaping perceptions of group belonging (Shamoa-Nir & Milford, 2025). Experimental studies demonstrate that even imagined intergroup contact enhances identification with a common ingroup by increasing perceived similarity and enabling social recategorization (Hu et al., 2025). And quantitative research in ethnically diverse university contexts in China indicates that intergroup contact significantly and positively predicts Chinese cultural identity (Quan et al., 2025).

Taken together, these findings suggest that intergroup contact, particularly when characterized by positive interaction quality, plays a crucial role in fostering cultural identity. Accordingly, the present study proposes the Hypothesis 1 (H1). Positive intergroup contact positively predicts Chinese cultural identity among Taiwanese students enrolled in universities in Mainland China.

### The mediating role of cultural adaptation

From the perspective of acculturation theory, individuals who engage in sustained intergroup interactions gradually adjust their social roles, interaction patterns, cultural behaviours, and everyday practices, thereby entering a process of cultural adaptation (Schwartz et al., 2010). Through this process, individuals reconstruct their understanding of the self and their relationship to the surrounding cultural environment (Ward & Szabó, 2023). Cultural adaptation is therefore often conceptualized as a key mechanism linking external social contact to internal identity formation, reflecting the transformation from situational interaction to psychological identification (Benet Martínez & Haritatos, 2005). However, the applicability of this framework to Taiwanese students enrolled in universities in Mainland China requires further qualification. Unlike typical immigrant or international student populations, these students are not entering a wholly unfamiliar cultural environment. Instead, they often possess a pre-existing level of identification with Chinese culture prior to their arrival (B. Zhang et al., 2022). In this context, cultural adaptation may be shaped less by differences in cultural symbols or traditions, and more by individuals’ interpretations of social norms, institutional arrangements, and patterns of social interaction (Zhuang, 2025). Such differences do not necessarily produce cultural distance in the conventional sense, but may introduce interpretive tensions in how shared cultural meanings are understood and enacted in everyday life.

Cultural adaptation in this study thus refers to the process through which Taiwanese students adjust their behavioural patterns, communication styles, and social participation in response to the Mainland Chinese social environment, while simultaneously reinterpreting culturally shared meanings within a distinct institutional and social context (Li, 2025). This process involves not only behavioural adjustment, but also the gradual alignment or negotiation of values, expectations, and social roles (Valladares & Taylor, 2025). Accordingly, cultural adaptation is operationalized as individuals’ degree of adjustment to local social norms, participation in campus and social life, and perceived ease of functioning within the broader social environment.

Existing research provides empirical support for the link between intergroup contact and cultural adaptation. Longitudinal studies have shown that positive intergroup contact can enhance individuals’ adaptive capacities by reducing uncertainty and facilitating social integration (Gebregergis & Csukonyi, 2025; Hai et al., 2025). Similarly, research on international students indicates that frequent and positive interactions with host-group members are associated with higher levels of cultural adaptation (T. Zhang & Ren, 2024). These findings suggest that intergroup contact provides the experiential basis through which individuals acquire context-specific knowledge and develop adaptive competencies. At the same time, although direct evidence linking cultural adaptation to cultural identity remains limited, a growing body of research points to a close association between the two constructs. For instance, acculturative stress has been found to negatively predict identity identification, implying that successful adaptation facilitates identity development (Malota & Mucsi, 2025). Other studies further conceptualize cultural adaptation as an integral component of cultural identity formation (Tang & Zhang, 2023; Wei, 2025).

Taken together, these arguments suggest that cultural adaptation functions not merely as a process of external adjustment, but as a mediating mechanism through which intergroup contact is translated into identity-related outcomes. In contexts characterized by shared cultural foundations but differing social and institutional environments, this process involves not only adaptation to new practices, but also the reinterpretation of culturally shared meanings. Accordingly, the present study proposes the Hypothesis 2 (H2): Cultural adaptation mediates the relationship between intergroup contact and Chinese cultural identity among Taiwanese students enrolled in universities in Mainland China.

### The moderating role of openness to experience

Openness to experience is a core dimension of the Big Five personality framework and refers to individuals’ tendencies to accept, explore, and engage with novel experiences, ideas, and cultural diversity at cognitive, affective, and behavioral levels (Poort et al., 2021). Individuals high in openness typically exhibit strong curiosity, cognitive flexibility, and tolerance for ambiguity (Bouazzaoui et al., 2024). In cross-cultural research, openness to experience has been widely recognized as an important individual-difference factor associated with cultural adaptation and identity development (Kosic, 2025; McCrae & Costa, 1997). However, beyond its direct effects, openness to experience may play a more critical role in shaping how individuals interpret and respond to intergroup contact (Yakunina et al., 2012). Rather than independently promoting cultural identity, openness influences the extent to which contact experiences are cognitively processed (DeYoung, 2015), emotionally internalized (McCrae, 2007), behaviorally enacted (LePine et al., 2000), thereby affecting whether and how such experiences are translated into identity-related outcomes (Schwartz et al., 2010). From a cognitive perspective, individuals high in openness are more likely to approach intergroup interactions with curiosity and a willingness to understand differences, which enhances empathic engagement and reduces defensive reactions (Yakunina et al., 2012). As a result, they are more inclined to interpret contact experiences as opportunities for learning and meaning-making, facilitating the integration of new cultural perspectives into their self-concept. Empirical evidence supports this mechanism. In a large-scale survey of Canadian and Norwegian university students, Poort et al. (2021) found that openness to experience was positively associated with deeper cognitive engagement in intergroup interactions. From a behavioral perspective, openness also influences individuals’ level of active participation in intergroup contexts. Individuals high in openness tend to engage more frequently and more extensively in social interactions, which increases both the quantity and quality of intergroup contact. For example, qualitative research on first-generation Chinese American immigrants shows that individuals with higher openness are more likely to participate in social activities and cultural exchanges, thereby facilitating the internalization of dominant cultural norms and values (X. Chen, 2025). Longitudinal evidence further indicates that openness to experience contributes to improvements in the quality of intergroup contact over time (Vezzali et al., 2018).

In contrast, individuals lower in openness may exhibit greater resistance to unfamiliar perspectives and a stronger tendency to maintain existing cognitive frameworks (Oreg, 2003), which can limit the depth of engagement in intergroup contact (Hodson, 2011) and constrain the extent to which such contact leads to identity change (Verkuyten, 2018). This distinction is particularly salient in contexts characterized by shared cultural foundations but differences in social norms and institutional arrangements, such as the experience of Taiwanese students in Mainland China. In such settings, where cultural familiarity coexists with interpretive differences, the transformation of contact experiences into cultural identification depends less on exposure itself and more on how individuals process and make sense of these experiences.

Building on these arguments, openness to experience can be understood as a key boundary condition that shapes the strength of the relationship between intergroup contact and cultural identity. Accordingly, the present study proposes Hypothesis 3 (H3). Openness to experience moderates the relationship between intergroup contact and Chinese cultural identity, such that the positive effect of intergroup contact on cultural identity is stronger among Taiwanese students enrolled in universities in Mainland China who exhibit higher levels of openness to experience.

Collectively, the proposed hypotheses outline an integrated framework in which intergroup contact influences Chinese cultural identity both directly and indirectly through cultural adaptation, while this process is further conditioned by individual differences in openness to experience. Specifically, intergroup contact provides the experiential basis for cultural adaptation, which in turn facilitates identity development, and the strength of this process varies depending on individuals’ level of openness, as it shapes how contact experiences are interpreted and internalized. A conceptual model illustrating all proposed hypotheses is presented in Figure 1.

**Figure 1.**
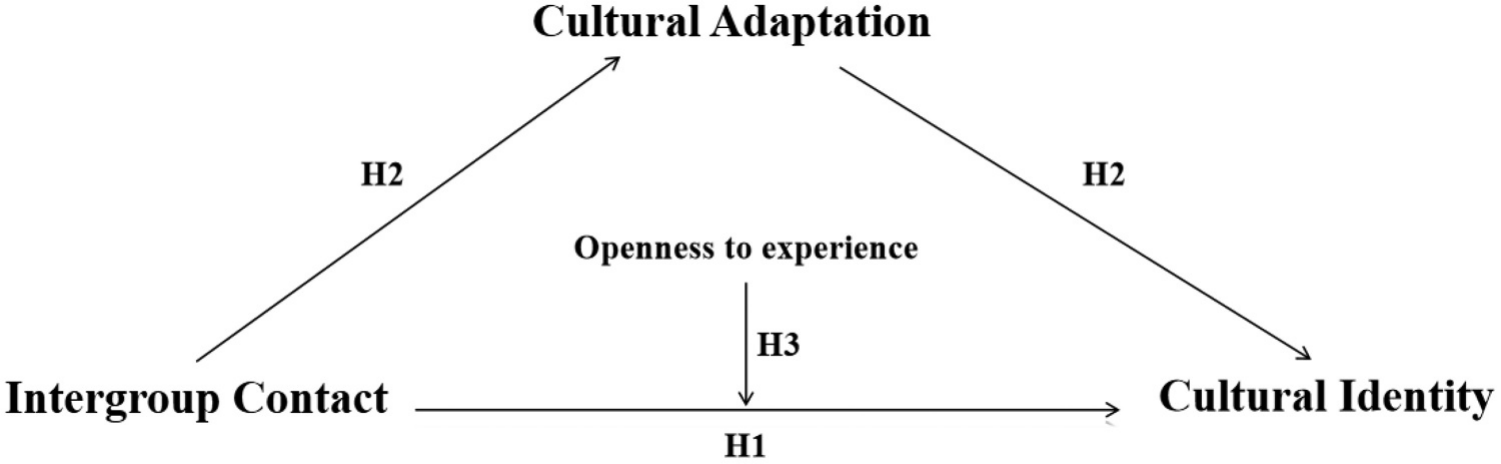
Proposed hypothesized model.

## Method

### Participants

An a priori power analysis was conducted using G*Power 3.1 to determine the required sample size. Assuming a medium effect size (*f* ^2^ = 0.15), a significance level of α = 0.05, statistical power of 0.95, and four predictors in a multiple linear regression model, the minimum required sample size was estimated to be 129 participants. Given the longitudinal design of the present study, which involved data collection at three time points, a certain degree of participant attrition was anticipated.

In this study, participants were recruited from three comprehensive universities located in Fujian and Guangdong provinces, both of which are regions characterized by relatively frequent cross-strait interactions and established channels for enrolling Taiwanese students. These universities are authorized to independently admit Taiwanese students and represent typical higher education institutions that host a stable population of cross-regional students. A convenience sampling strategy was adopted, and participants were recruited offline with the assistance of faculty members at the respective institutions. All participants had no prior experience of living in Mainland China before entering university. Although detailed institutional statistics (e.g., exact proportions of Taiwanese students) were not available, the selected universities provide relevant and contextually appropriate settings for examining intergroup contact and cultural identity among Taiwanese students in Mainland China.

Drawing on prior research regarding the timing of survey waves (Cao et al., 2024), the first wave of data collection was conducted in March 2025, with a total of 347 participants completing the survey. Due to reasons such as leave of absence or suspension of study, 21 participants were lost at the second wave (June 2025), and an additional 27 participants were lost at the third wave (September 2025), resulting in a total attrition of 48 participants and an attrition rate of 13.83%. After excluding 13 cases with invalid data – defined as missing responses exceeding 20%, identical responses across all items, failure on attention check items, or excessively short completion times – a final sample of 286 participants with valid data across all three waves was retained for subsequent analyses, yielding an effective retention rate of 82.42%. The final sample consisted of 130 male and 156 female participants, with a mean age of 18.99 years (*SD* = 1.16). This study was reviewed and approved by the Ethics Committee of the School of Psychology at Fujian Normal University. All participants were at least 18 years of age. Prior to participation, all participants provided written informed consent after being fully informed about the purpose and procedures of the study. Participation was entirely voluntary, and confidentiality was assured, with no personally identifiable information collected. Upon completion of all three waves of data collection, participants received monetary compensation of 50 RMB for their participation.

Detailed demographic characteristics of the participants are presented in Table 1. Attrition analyses indicated no significant differences between retained and attrited participants in terms of gender, χ^2^(1) = 2.65, *p* = 0.104, or cultural identity measured at Time 1, *t*(345) = 0.28, *p* = 0.78.

**Table 1.** Demographic information of participants (*N* = 345).

Type	*n*	*n* (%)
Male	130	45.45%
Female	156	54.55%
**Grade**		
First-year University	84	23.37%
Second-year University	63	22.03%
Third-year University	78	27.27%
Fourth-year University	61	21.33%
**Major Distribution**		
Arts	121	42.31%
Sciences	165	57.69%

### Measures

#### Intergroup contact

Intergroup contact was measured using the Intergroup Contact Questionnaire, which has demonstrated good reliability and validity in previous research (L. Chen & Hong, 2025; Shi et al., 2014). The scale consists of two dimensions: contact quantity and contact quality, with four items in each dimension, yielding a total of 8 items. All items were rated on a 7-point Likert scale ranging from 1 (strongly disagree) to 7 (strongly agree), with higher scores indicating higher levels of intergroup contact. In the present study, the Cronbach’s α coefficients were 0.94 for the contact quantity subscale and 0.91 for the contact quality subscale, indicating excellent internal consistency. Following established practice in prior studies, an overall intergroup contact index was computed by multiplying contact quantity and contact quality scores, with higher values reflecting higher levels of intergroup contact (Korol et al., 2018).

#### Cultural adaptation

Cultural adaptation was assessed using the Cross-Cultural Adaptation Scale, which has demonstrated satisfactory reliability and validity (Kuang & Wu, 2019). The scale comprises two dimensions – sociocultural adaptation and psychological adaptation – and includes a total of eight items. Responses were recorded on a 5-point Likert scale ranging from 1 (strongly disagree) to 5 (strongly agree). Higher scores indicate better levels of cultural adaptation. In the current sample, the scale exhibited excellent internal consistency, with a Cronbach’s α coefficient of 0.93.

#### Chinese cultural identity

Chinese cultural identity was measured using the Chinese Cultural Identity Scale developed for Hong Kong, Macao, and Taiwanese students studying at universities in Mainland China (Sun, 2023). The scale consists of three dimensions: cultural cognitive preference, cultural affect, and cultural practice, comprising a total of 19 items. Participants responded using a 5-point Likert scale ranging from 1 (strongly disagree) to 5 (strongly agree), with higher scores indicating stronger Chinese cultural identity. In the present study, the scale demonstrated excellent internal consistency, with a Cronbach’s α coefficient of 0.92, indicating good reliability and construct validity.

#### Openness to experience

Openness to experience was measured using the Openness subscale of the Big Five Inventory (Gong et al., 2023; John & Srivastava, 1999). This subscale consists of 10 items rated on a 5-point scale ranging from 0 (strongly disagree) to 4 (strongly agree). Higher scores reflect higher levels of openness to experience. In the present study, the Openness subscale demonstrated excellent internal consistency, with a Cronbach’s α coefficient of 0.94.

### Procedure

Following prior research on time-lagged survey designs and analytical strategies (Liao et al., 2025), the present study employed a three-wave time-lagged survey design over a six-month period. Data were collected at three time points: March 2025 (Time 1), June 2025 (Time 2), and September 2025 (Time 3), with approximately three-month intervals between waves. The three-month interval was selected based on both theoretical and empirical considerations. From a theoretical perspective, cultural adaptation and identity development are dynamic processes that unfold over time rather than occurring instantaneously (Ward & Geeraert, 2016). Prior research has shown that meaningful changes in sociocultural adaptation often occur during the initial months of cross-cultural transition (Ward & Kennedy, 1999). From an empirical perspective, previous studies on international students’ cross-cultural adjustment have adopted similar three-wave designs with approximately three-month intervals (Cao et al., 2024), suggesting that this time frame is sufficient to capture short-term changes in adaptation-related processes while minimizing participant attrition.

At Time 1, participants completed measures of intergroup contact, openness to experience, Chinese cultural identity, and demographic information. At Time 2, cultural adaptation was assessed as an intermediate process variable. At Time 3, Chinese cultural identity was measured again to capture subsequent identity outcomes. All survey administrations were scheduled on weekend mornings at 10:00 a.m. At each wave, questionnaires were distributed via an online survey platform, and participants completed the surveys independently.

Cultural adaptation was measured only at Time 2 because it was conceptualized as a mediating mechanism linking earlier intergroup contact to later cultural identity. However, this design does not allow for the examination of changes in cultural adaptation over time or its autoregressive effects. Therefore, the present study provides time-lagged evidence consistent with the proposed mediation process, rather than a full test of longitudinal mediation.

## Results

### Common method bias test

All data analyses were conducted using SPSS 26.0. Descriptive statistics, correlation analyses, and tests of the moderated mediation model were performed. Prior to hypothesis testing, common method bias was assessed. First, Harman’s single-factor test was applied to all variables measured at Time 1. Exploratory factor analysis revealed six factors with eigenvalues greater than 1 across the 39 items, and the first factor accounted for 31.76% of the total variance, which is below the commonly accepted threshold of 40%. These results suggest that common method variance was not a serious concern in the present study.

To further ensure methodological rigor, a more stringent test was conducted using confirmatory factor analysis (CFA). Specifically, a single-factor model was constructed in which all measurement items across the three waves (T1, T2, and T3) were specified to load onto a single common method factor. The model demonstrated a poor fit to the data (χ^2^/*df* = 18.31, CFI = 0.556, TLI = 0.467, RMSEA = 0.179, SRMR = 0.187), indicating that the single-factor solution did not adequately represent the data structure. Taken together, these findings provide robust evidence that common method bias did not substantially influence the results of the present study.

### Discriminant validity between cultural adaptation and cultural identity

To further examine the empirical distinctiveness of cultural adaptation and cultural identity, we conducted supplementary CFAs comparing a two-factor model with a one-factor model. The two-factor model showed substantially better fit than the one-factor model, χ^2^/*df* = 3.88, *p*  < 0.001, CFI = 0.809, TLI = 0.784, RMSEA = 0.109, SRMR = 0.108, versus χ^2^/*df* = 6.95, *p*  < 0.001, CFI = 0.575, TLI = 0.539, RMSEA = 0.144, SRMR = 0.150. Moreover, the estimated correlation between the two latent factors was *r* = 0.463 (*p* < 0.001). These results suggest that cultural adaptation and cultural identity are meaningfully related yet empirically distinguishable.

### Descriptive statistics and correlations

As shown in Table 2, the key variables measured at Time 1 (T1), Time 2 (T2), and Time 3 (T3) were significantly correlated with one another. These correlations provide preliminary support for the hypothesized relationships among the study variables. In subsequent analyses, baseline cultural identity at Time 1 and gender were included as covariates.

**Table 2.** Descriptive statistics and correlations of variables.

	*M*	*SD*	1	2	3	4	5
1. T1 Intergroup Contact	473.07	217.11					
2. T2 Cultural Adaptation	37.45	6.59	0.71**				
3. T3 Cultural Identity	78.43	11.16	0.56**	0.63**			
4. T1 Openness to Experience	37.07	8.07	0.63**	0.57**	0.46**		
5. T1 Cultural Identity	71.82	12.62	0.38**	0.51**	0.68**	0.45**	
6. Gender	0.53	0.50	0.01	0.03	0.04	0.05	0.04

Note: Male = 1, female = 0. *p* < 0.05 = *; *p* < 0.01 = **; *p* < 0.001 = ***. The same notation is used throughout.

### Testing the indirect effect through cultural adaptation

Prior to the mediation analysis, all variables except gender were standardized. The indirect effect through cultural adaptation was examined using the PROCESS macro for SPSS (Version 3.5, Model 4). Baseline cultural identity at Time 1 and gender were included as covariates, and the full regression results are reported in Table 3. The results indicated that, after controlling for the covariates, intergroup contact significantly and positively predicted cultural adaptation (B = 0.70 for path a, *p*  < 0.001). Gender was significantly associated with cultural adaptation, whereas baseline cultural identity at Time 1 was not. When both intergroup contact and cultural adaptation were simultaneously entered into the regression equation predicting cultural identity, intergroup contact remained a significant positive predictor of cultural identity (direct effect, B = 0.24 for path c’, *p*  < 0.001), and cultural adaptation also significantly and positively predicted cultural identity (B = 0.46 for path b, *p*  < 0.001). In this equation, baseline cultural identity at Time 1 was not significantly associated with cultural identity, and gender showed only a marginally small association with cultural identity. Bootstrap analyses (5,000 resamples) further demonstrated that the indirect effect of intergroup contact on cultural identity via cultural adaptation was significant (path ab = 0.32, 95% CI [0.23, 0.42]). The indirect effect accounted for 57.28% of the total effect, as indicated by the ratio ab/(ab + c′). Further details are provided in Table 4.

**Table 3.** Regression results for the mediation model and bootstrap estimates of effects.

Dependent Variable	Predictor	*B*	*SE*	*t*	*p*
T2 Cultural Adaptation	T1 Intergroup Contact	0.70	0.04	16.66	<0.001
	T1 Cultural Identity	−0.06	0.04	−1.51	0.133
	Gender	−0.09	0.09	−2.19	0.029
T3 Cultural Identity	T1 Intergroup Contact	0.24	0.06	3.77	<0.001
	T2 Cultural Adaptation	0.46	0.06	7.18	<0.001
	T1 Cultural Identity	−0.05	0.05	−1.09	0.279
	Gender	0.09	0.09	1.98	0.049

**Table 4.** Bootstrap estimates of the total, direct, and indirect effects of intergroup contact on cultural identity.

Type of Effect	Effect Value	Standard Error	95%CI	Proportion of Total Effect
Total Effect	0.56	0.05	[0.46，0.66]	100%
Direct Effect	0.24	0.06	[0.11，0.36]	42.72%
Indirect Effect	0.32	0.05	[0.23，0.42]	57.28%

### Testing the moderating role of openness to experience

To further examine the mediating role of cultural adaptation and the potential moderating effect of openness to experience, Model 5 of the PROCESS macro for SPSS (Version 3.5) was employed. Baseline cultural identity at Time 1 and gender were included as covariates. The results of the moderated mediation analysis are presented in Table 5 and Figure 2. Intergroup contact significantly and positively predicted cultural adaptation, explaining 50.24% of the variance in cultural adaptation (*R*^2^ = 0.50). Baseline cultural identity at Time 1 was not significantly associated with cultural adaptation, whereas gender showed a significant association.

**Figure 2.**
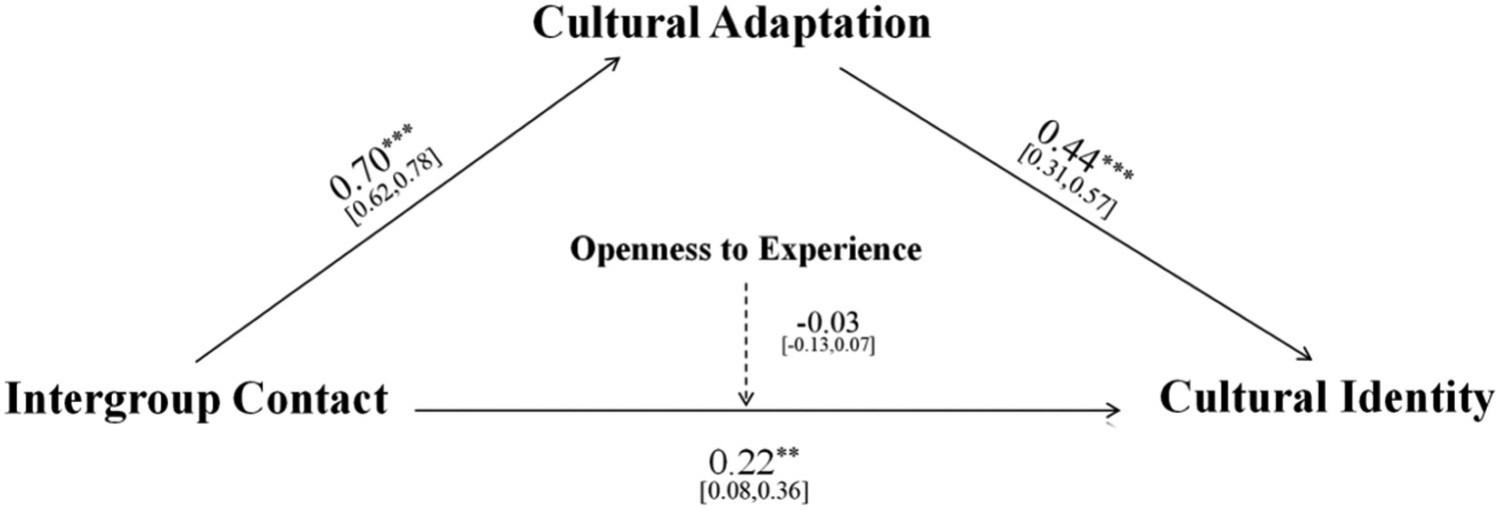
The mechanism of intergroup contact on cultural identity.

**Table 5.** Results of the moderated mediation effect test.

Regression Equation		Model Fit					
Dependent Variable	Independent Variable	*R*^2^	*B*	*SE*	*t*	*p*	95% CI
T2 Cultural Adaptation	T1 Intergroup Contact	0.50	0.70	0.04	16.66	<0.001	[0.62, 0.78]
	T1 Cultural Identity		−0.06	0.04	−1.51	0.133	[−0.15, 0.02]
	Gender		−0.19	0.09	−2.19	0.029	[−0.35, −0.02]
T3 Cultural Identity	T1 Intergroup Contact	0.43	0.22	0.07	3.04	0.003	[0.08, 0.36]
	T2 Cultural Adaptation		0.44	0.07	6.58	<0.001	[0.31, 0.57]
	T1 Openness to Experience		0.07	0.06	1.08	0.281	[−0.05, 0.19]
	T1 Intergroup Contact × T1 Openness to Experience		−0.03	0.05	−0.62	0.536	[−0.13, 0.07]
	T1 Cultural Identity		−0.04	0.05	−0.88	0.382	[−0.13, 0.05]
	Gender		0.18	0.09	1.97	0.050	[0.00, 0.36]

Note: All continuous variables were standardized prior to analysis; gender was entered as a binary covariate. Baseline cultural identity at Time 1 and gender were included as covariates in all equations.

When cultural identity was entered as the dependent variable, intergroup contact remained a significant positive predictor, and cultural adaptation also significantly predicted cultural identity. Openness to experience was not significantly associated with cultural identity, and the interaction between intergroup contact and openness to experience was not significant. In this model, baseline cultural identity at Time 1 was not significantly associated with cultural identity, whereas the coefficient for gender was positive and at the threshold of statistical significance. The overall model accounted for 43.28% of the variance in cultural identity (*R*^2^ = 0.43).

The conditional direct effects of intergroup contact on cultural identity were significant at low (*B* = 0.25, 95% CI [0.06, 0.43]), mean (*B* = 0.22, 95% CI [0.08, 0.36]), and high (*B* = 0.19, 95% CI [0.03, 0.34]) levels of openness to experience. In addition, the indirect effect of intergroup contact on cultural identity through cultural adaptation remained significant (effect = 0.31, 95% CI [0.21, 0.41]). Taken together, these findings were consistent with an indirect effect through cultural adaptation but do not support a moderating effect of openness to experience on the direct association between intergroup contact and cultural identity.

Although the model explained a relatively substantial proportion of variance in cultural adaptation (*R*^2^ = 0.50) and cultural identity (*R*^2^ = 0.43), these values should be interpreted with caution. The relatively high explained variance may be attributable, in part, to the conceptual proximity among the constructs examined within the acculturation framework. Additionally, although temporal separation across waves was implemented to mitigate common method bias, the use of self-report measures may still have contributed to inflated associations among variables.

## Discussion

Given the inconsistent findings in prior research on Chinese cultural identity among Taiwanese youth, the present study is the first to adopt a longitudinal design to examine the influence of intergroup contact on Chinese cultural identity among Taiwanese students enrolled in universities in Mainland China, from a micro-level individual perspective. By tracking participants over three time points, this study provides a dynamic examination of how intergroup contact shapes cultural identity and helps to clarify possible underlying processes. The results demonstrated that intergroup contact positively predicted Chinese cultural identity among Taiwanese students enrolled in universities in Mainland China. Moreover, cultural adaptation showed a significant indirect effect in this relationship, whereas the moderating effect of openness to experience was not supported. In the following sections, these findings are discussed in detail in light of existing theoretical frameworks and empirical evidence.

### The promoting effect of intergroup contact on Chinese cultural identity

The present study found that positive intergroup contact significantly promotes Chinese cultural identity among Taiwanese students enrolled in universities in Mainland China. This finding is consistent with prior research conducted by scholars in Mainland China (Lian et al., 2025; Ye et al., 2025), supporting the view that contact facilitates identity formation. At the same time, the present results add new longitudinal empirical evidence to the literature on cultural identity among youth across the Taiwan Strait.

Notably, this finding differs from conclusions reported in some Taiwan-based studies, which have suggested that intergroup contact does not necessarily promote cultural identity. We argue that this discrepancy may be explained by three key factors. First, the participants in the present study were Taiwanese students enrolled in universities in Mainland China, a population that differs substantially from Taiwanese youth residing solely in Taiwan who lack sustained experiences of studying and living in Mainland China. Living, studying, and socializing with Mainland Chinese peers provides these students with a form of intergroup contact that is authentic, continuous, and in-depth, far exceeding the scope of short-term exchanges, academic visits, or incidental interactions. As a result, they are more likely to engage in multiple dimensions of contact and adaptation, including cultural practices, daily interactions, language use, and social integration. The quality and depth of such contact are therefore considerably higher than those experienced by Taiwanese youth whose exposure is limited to brief or superficial interactions. This high-intensity, multidimensional, and long-term contact is more conducive to the construction of Chinese cultural identity. Second, the present study incorporated cultural adaptation as a key explanatory variable and found a significant indirect effect through cultural adaptation between intergroup contact and cultural identity. In other words, effective intergroup contact is not merely superficial interaction but operates by facilitating individuals’ comprehensive adaptation to mainstream cultural norms, behaviours, social roles, and customs. Through this adaptive process, individuals gradually integrate into social practices, thereby enhancing their sense of belonging and cultural identity. In contrast, studies that focus solely on contact without accounting for adaptation may fail to detect changes in identity. Some previous Taiwan-based studies may have reached the conclusion that contact does not equal identity promotion due to sample limitations – such as focusing on short-term exchange students or occasional interactions – or because they measured intergroup contact without assessing cultural adaptation.

Finally, the broader sociocultural context in which the present study was conducted may provide institutional and structural conditions that facilitate identity formation. Campus-based teaching, student organizations, and everyday interactions in Mainland Chinese universities offer Taiwanese students continuous and authentic opportunities for cultural contact and cultural experience. These contextual factors may create an environment that is more conducive to identity transformation. In contrast, Taiwanese youth whose contact experiences are fragmented, temporary, or superficial may lack sufficient exposure to trigger deeper reconstruction of cultural identity.

Beyond these contextual explanations, the present findings also carry important theoretical implications. This study not only confirms the basic logic of intergroup contact in promoting social identity reconstruction, but more importantly extends the applicability of intergroup contact theory and the Common Ingroup Identity Model to the cross-strait cultural context. Previous research has primarily examined how positive intergroup contact fosters more inclusive social identities in Western multiethnic societies, whereby individuals transcend original group boundaries and expand their self-concept to a higher-order common group through intergroup interaction (Reimer et al., 2020). The present results further demonstrate that within the context of Chinese culture and cross-strait social relations, long-term and high-quality intergroup contact can similarly be associated with processes of identity reconstruction. This finding not only enriches the cultural generalizability of intergroup contact theory but also provides new empirical support for extending the “contact–identity” pathway to East Asian sociocultural contexts.

### Cultural adaptation as an indirect link between intergroup contact and Chinese cultural identity

The present study found evidence consistent with a significant indirect effect through cultural adaptation in the relationship between intergroup contact and Chinese cultural identity. Intergroup contact not only directly predicted cultural identity, but was also indirectly associated with cultural identity through cultural adaptation. Notably, the indirect effect via cultural adaptation accounted for 57.28% of the total effect. This substantial proportion suggests that cultural adaptation may be an important process linking intergroup contact to cultural identity among Taiwanese students enrolled in universities in Mainland China.

This finding is consistent with recent research on cross-cultural psychological mechanisms, which emphasizes cultural adaptation as a critical pathway through which cross-cultural contact influences identity reconstruction (Ward & Szabó, 2023). Through adaptive processes involving participation in social practices, individuals gradually develop new cultural identity structures. Mere contact alone is insufficient to automatically promote identity change; rather, meaningful transformation of cultural identity may be more likely when individuals actively engage with the mainstream culture, comprehend its value system, and translate this understanding into adaptive everyday behaviors.

Importantly, the present findings also contribute to a growing body of research on culturally similar but institutionally divided groups. In contexts such as East and West Germany after reunification, or North and South Korea, scholars have documented that shared cultural heritage does not necessarily translate into immediate psychological identification, as prolonged institutional and ideological differences can shape distinct social norms, values, and identity orientations (Becker et al., 2020). These studies suggest that even among culturally homologous populations, identity convergence requires more than contact – it requires processes of adaptation to everyday practices and social systems. In this regard, the present study extends this line of research by showing that cultural adaptation may be an important process through which contact is linked to identity reconstruction in culturally related but institutionally differentiated contexts, such as cross-strait student mobility.

Moreover, the significant indirect effect through cultural adaptation further suggests that cultural adaptation may function as an important link, rather than a peripheral or supplementary factor, between intergroup contact and cultural identity (H. Zhang & Wang, 2024). For Taiwanese students enrolled in universities in Mainland China, long-term residence in a highly interactive and integrative university environment entails not only frequent interactions with Mainland Chinese peers, but also deep adaptation to academic systems, social norms, and daily life practices. This depth of sociocultural embeddedness amplifies the influence of intergroup contact on psychological identification, such that identity formation is more likely to occur through a pathway of adaptation, internalization, and re-identification, rather than being directly triggered by surface-level contact experiences alone.

At a conceptual level, the strong mediating effect observed in this study also raises important questions regarding the relationship between cultural adaptation and cultural identity. In cross-cultural contexts, adaptation to everyday practices, norms, and social expectations often provides an important basis for the development of cultural belonging and identification. At the same time, CFA results suggested that these two constructs are related but empirically distinguishable. Thus, the present findings support viewing cultural adaptation not as interchangeable with cultural identity, but as an important pathway through which intergroup contact contributes to identity formation.

In contrast, when intergroup contact lacks the support of cultural adaptation, superficial or fragmented interactions are unlikely to generate profound changes in cultural identity. This mechanism helps to explain why some Taiwan-based studies have reported that contact does not necessarily promote identity (H. Lin, 2023). Finally, the present findings not only highlight the important role of cultural adaptation in the process through which intergroup contact influences cultural identity, but also suggest the stage-like nature of identity formation. Identity construction in cross-cultural contexts often involves a gradual internalization process progressing from behavioural and social adaptation to psychological identification, with cultural adaptation serving as a possible foundation for identity reconstruction (T. Zhang & Ren, 2024). The present results indicate that adaptation at the behavioural and social practice levels not only helps Taiwanese students reduce feelings of cultural alienation, but also enhances their sense of cultural belonging and value congruence, thereby facilitating the internalization of Chinese cultural identity. These findings provide empirical support for cross-cultural adaptation theory and offer practical implications for practical initiatives aimed at promoting cross-strait youth integration (Xiao & Li, 2024).

An additional finding worth noting is that gender, although included only as a control variable, showed a significant association with cultural adaptation and a comparatively weak association with cultural identity. Given that gender was coded as 1 = male and 0 = female, the negative coefficient for gender in the cultural adaptation model suggests that male students reported lower levels of cultural adaptation than female students. This pattern is broadly consistent with prior research indicating that female students often display stronger sociocultural adjustment and are more likely to seek interpersonal support in new environments, which may facilitate adaptation to unfamiliar social and institutional contexts (Güngör & Bornstein, 2013; Kocaturk et al., 2026). By contrast, the positive coefficient for gender in the cultural identity model suggests that male students showed slightly higher Chinese cultural identity than female students, although this effect was relatively small and should be interpreted cautiously given that it only reached the conventional threshold of statistical significance. One possible explanation is that cultural adaptation and cultural identity, while related, do not necessarily develop in identical ways. Adaptation reflects adjustment to everyday practices and social environments, whereas identity involves a broader sense of symbolic belonging and self-definition (Berry, 1997; Ward & Szabó, 2023). Since gender was not a focal variable in the present study, these findings should be regarded as supplementary rather than conclusive. Future research could examine more directly whether gender differences in social interaction patterns, perceived support, or identity-related meaning-making contribute to distinct trajectories of adaptation and cultural identity among Taiwanese students in Mainland China.

### The non-significant moderating effect of openness to experience

Although prior research has suggested that openness to experience can facilitate individuals’ engagement in novel cultural environments and promote cross-cultural adaptation, the present study did not find a significant moderating effect of openness to experience on the relationship between intergroup contact and Chinese cultural identity.

Importantly, this null finding should be interpreted with caution. First, interaction effects are typically more difficult to detect than main effects and often require substantially larger sample sizes to achieve adequate statistical power (Aguinis et al., 2005). Although the present study was sufficiently powered to detect medium-sized main effects, it may have been underpowered to detect relatively small interaction effects. Therefore, the absence of a significant moderation effect does not necessarily imply the absence of a true effect.

Second, measurement characteristics may have attenuated the moderating effect. Openness to experience was assessed as a broad personality trait (Soto, 2016), which may not be sufficiently sensitive to capture context-specific variability in cross-cultural engagement. Prior research has suggested that more proximal constructs, such as cultural intelligence or intercultural competence, may serve as stronger moderators in cross-cultural contexts (Ahadi & Puente-Díaz, 2011; Jurásek & Wawrosz, 2023; Shu et al., 2017). In this sense, openness may be too distal a dispositional variable to condition whether intergroup contact is translated into cultural identity under a specific educational and sociocultural context. Rather than directly moderating this pathway, its influence may be partially channeled through more context-relevant capacities, such as the ability to interpret cultural cues, regulate uncertainty, or engage effectively in intercultural interaction. Future research could therefore examine whether more domain-specific individual difference variables yield stronger moderating effects.

Third, alternative model specifications may provide additional insights into the role of openness. Rather than functioning as a moderator, openness to experience may operate as an antecedent or correlate of intergroup contact and cultural adaptation, or exert indirect effects through more proximal psychological mechanisms. This possibility may be especially relevant in the present study, because openness could play a stronger role in the earlier selection stage – namely, the decision to pursue higher education in Mainland China – than in the later stage of shaping how intergroup contact relates to cultural identity once students are already embedded in that environment. From this perspective, openness may influence entry into cross-cultural settings more strongly than the downstream conversion of contact experiences into identity-related outcomes. Future studies could test alternative models, such as moderated mediation at different stages or models in which personality traits influence exposure to or quality of intergroup contact.

Finally, contextual characteristics of the sample may have contributed to the non-significant moderation effect. A possible selection effect in the present sample should be considered when interpreting this null finding. Because participants voluntarily chose to pursue higher education in Mainland China (Birtel et al., 2024), they may have had a relatively higher baseline level of openness to experience than the broader population of Taiwanese youth. If openness was already elevated and less variable in this cohort, range restriction may have attenuated the estimated interaction effect. Similarly, limited variability in openness within this self-selected sample may have reduced its ability to differentiate individuals in the contact–identity link. This possibility suggests that the non-significant moderation result should not be interpreted as conclusive evidence that openness is irrelevant to cross-cultural processes. Rather, it may indicate that the influence of openness depends on both sample composition and contextual conditions.

At a theoretical level, this finding may suggest that the educational context examined here was a relatively strong situation. Institutional arrangements, shared academic demands, and recurring opportunities for contact may have limited the extent to which broad personality differences shaped identity-related outcomes. When intercultural encounters are embedded in structured environments with relatively clear role expectations and repeated interaction, contextual factors may exert a stronger influence on adaptation and identification processes, thereby reducing the observable moderating role of personality traits. In such settings, the extent to which intergroup contact contributes to cultural identity may depend less on openness itself than on the quality, meaning, and emotional tone of contact experiences, as well as on whether students feel accepted, understood, and able to integrate these experiences into a coherent sense of self.

However, rather than concluding that contextual factors dominate personality effects, the present findings suggest that the moderating role of openness may be contingent upon both situational strength and measurement specificity, highlighting the need for more nuanced person–situation interaction models in cross-cultural research. Overall，the non-significant moderating effect of openness does not necessarily weaken the theoretical integration with personality frameworks, but instead helps to delineate the boundary conditions under which personality traits influence cross-cultural processes. Taken together, the present results suggest that openness may matter, but not always in the same way, at the same stage, or with the same strength across different cross-cultural contexts. From a practical perspective, these findings suggest that interventions aimed at promoting cultural identity may benefit not only from fostering individual openness, but also from optimizing structural and contextual conditions that facilitate meaningful cultural engagement.

### Limitations and future directions

Despite the contributions of the present study, several limitations should be acknowledged. One important limitation concerns the sampling strategy. The sample was drawn from universities located in Fujian and Guangdong provinces, which are regions characterized by relatively high concentrations of Taiwanese students and frequent cross-strait interactions. While these settings provide a meaningful context for examining intergroup contact and cultural identity, the relatively homogeneous regional and institutional characteristics may limit the generalizability of the findings. More specifically, Taiwanese students in Fujian and Guangdong – particularly in Fujian – may experience cultural adaptation under conditions shaped by greater geographical proximity, closer dialectal or linguistic connections, and stronger cultural continuity with Taiwan. As a result, the adaptation process observed in the present sample may differ in important ways from that of Taiwanese students studying in other parts of Mainland China, such as northern metropolitan areas including Beijing and Shanghai. In addition, because participants were recruited from a limited number of universities, the findings may also reflect institution-specific contextual features. Future research is encouraged to include more diverse samples across different regions and institutional contexts to enhance external validity and to examine whether the observed relationships are robust across different regional settings. Another limitation relates to the research design. Although the present study employed a three-wave time-lagged design to introduce temporal separation among variables, it does not constitute a full longitudinal design. Therefore, the findings should be interpreted as providing time-lagged evidence consistent with the proposed mediation process, rather than a rigorous test of longitudinal mediation. Future studies could adopt fully longitudinal designs with repeated measurements of all key variables to better capture the dynamic processes of cultural adaptation and identity development. A further limitation concerns the reliance on self-report measures. Although procedural and statistical remedies were implemented, common method bias cannot be entirely ruled out. Future research could incorporate multi-method approaches, such as peer reports, behavioural indicators, or implicit measures, to enhance the robustness of the findings.

In addition, issues related to conceptual overlap warrant careful consideration. Although CFA results indicated that cultural adaptation and cultural identity were empirically distinguishable, the absolute fit of the two-factor model was not ideal. This suggests that some degree of conceptual overlap or measurement complexity may remain. In some theoretical perspectives, elements of cultural adaptation – particularly sociocultural competence – are treated as components of broader identity constructs. As such, the observed mediating effect may partly reflect structural proximity between constructs rather than a purely sequential process. Future research could address this issue by employing more fine-grained measurement strategies, testing discriminant validity more rigorously, or applying alternative analytical approaches such as network analysis to disentangle the structural relationships among adaptation and identity components. In addition, because the sample consisted of Taiwanese students who voluntarily chose to study in Mainland China, some degree of selection bias and possible range restriction in openness to experience cannot be ruled out. This may have reduced variability in openness to experience and constrained the detection of its moderating effect. Finally, although gender emerged as a significant covariate in some models, the present study was not designed to test gender differences systematically. Future studies could investigate whether these associations are robust and whether gender plays a more substantive role in cross-cultural adaptation and identity formation among Taiwanese students in Mainland China.

Overall, these considerations underscore the importance of integrating more rigorous longitudinal designs, multi-method measurements, and refined conceptual models in future research to further advance understanding of cross-cultural contact and identity formation processes.

## Conclusion

The present study yielded the following main conclusions: (1) Intergroup contact significantly promotes Chinese cultural identity among Taiwanese students enrolled in universities in Mainland China. (2) Cultural adaptation showed a significant indirect effect in the relationship between intergroup contact and Chinese cultural identity. (3) Openness to experience does not significantly moderate the effect of intergroup contact on Chinese cultural identity.

## Data Availability

The data that support the findings of this study are available from the corresponding author upon reasonable request.
